# Noninvasive monitoring of bilirubin photoisomer excretion during phototherapy

**DOI:** 10.1038/s41598-022-16180-9

**Published:** 2022-07-12

**Authors:** Yumiko Uchida, Yukihiro Takahashi, Yukihiro Morimoto, Peter Greimel, Asako Tosaki, Akiko Kumagai, Toshiya Nishikubo, Atsushi Miyawaki

**Affiliations:** 1 Division of Neonatal Intensive Care, Maternal, Fetal and Neonatal Medical Center, Nara Medical University Hospital, 840 Shijo-cho, Kashihara, Nara 634-8521 Japan; 2 R&D Division, Ushio Inc., Himeji, Japan; 3 SANKEN, Osaka University, Osaka, Japan; 4 Cell Function Dynamics, Brain Science Institute, RIKEN, Wako, Japan

**Keywords:** Biochemistry, Biological techniques, Biomarkers

## Abstract

Lumirubin is the most prevalently excreted hydrophilic bilirubin photoisomer in phototherapy for neonatal jaundice caused by excess hydrophobic unconjugated bilirubin (ZZ-bilirubin). We developed a simple method to estimate the amount of lumirubin by monitoring the reverse photoisomerization of lumirubin to ZZ-bilirubin. Although lumirubin formation was long considered irreversible, exposure to blue light in the presence of the fluorescent protein UnaG, which binds specifically and tightly to ZZ-bilirubin, enables the reverse photoisomerization of lumirubin. This reaction was first detected using a fluorescence assay of neonatal urine sampled during phototherapy and purified lumirubin. The phenomenon of reverse photoisomerization of lumirubin was validated using liquid chromatography–mass spectrometry, which confirmed that lumirubin is reconverted to ZZ-bilirubin in the presence of UnaG. Analyses of 20 urine samples from 17 neonates revealed a significant correlation (correlation coefficient [*r*] = 0.978; 95% confidence interval 0.867–0.979; *P* < .001) between lumirubin and ZZ-bilirubin concentration before and after reverse photoisomerization. In general, the rate of photo-reconversion of lumirubin to ZZ-bilirubin is approximately 40%. In conclusion, we demonstrate here that lumirubin can be photo-reconverted to ZZ-bilirubin via exposure to blue light in the presence of UnaG. Utilizing this approach, urinary lumirubin levels can be estimated using an easy-to-perform fluorescence assay.

## Introduction

UnaG, a protein derived from Japanese eel, was first cloned in 2013 and reported to exhibit high affinity and specificity for ZZ-bilirubin (ZZ-BR)^1^. The UnaG-ZZ-BR complex exhibits concentration-dependent fluorescence suitable for quantifying ZZ-BR even in the presence of albumin while requiring only a small amount of sample compared with the conventional method^1^.

Noninvasive phototherapy (PT) to reduce the body burden of unconjugated bilirubin has been widely used for nearly 6 decades for the treatment of neonatal hyperbilirubinemia^2–4^. Formation of the structural bilirubin photoisomer lumirubin (LR; EZ-cyclobilirubin) is thought to be an important route for bilirubin elimination during PT^5^. Despite the importance of LR, the clinical efficacy of LR measurement has not been assessed because high-performance liquid chromatography (HPLC) is the gold standard method for LR determination. This is because the measurement of LR by HPLC is accurate and quantitative, but the equipment is expensive, requires delicate handling, and takes time to measure^6,7^.

In the present study, we investigated the utility of estimating LR using a fluorescence assay, which is more convenient and easier to perform than HPLC analyses. As an LR-containing biological material that can be obtained noninvasively, urine samples were collected from premature infants undergoing PT for neonatal hyperbilirubinemia. Purified LR for comparison was obtained using a recently reported preparative thin-layer chromatography technique^8,9^.

## Results

### Fluorescence assay (FA)

**Figure 1:**
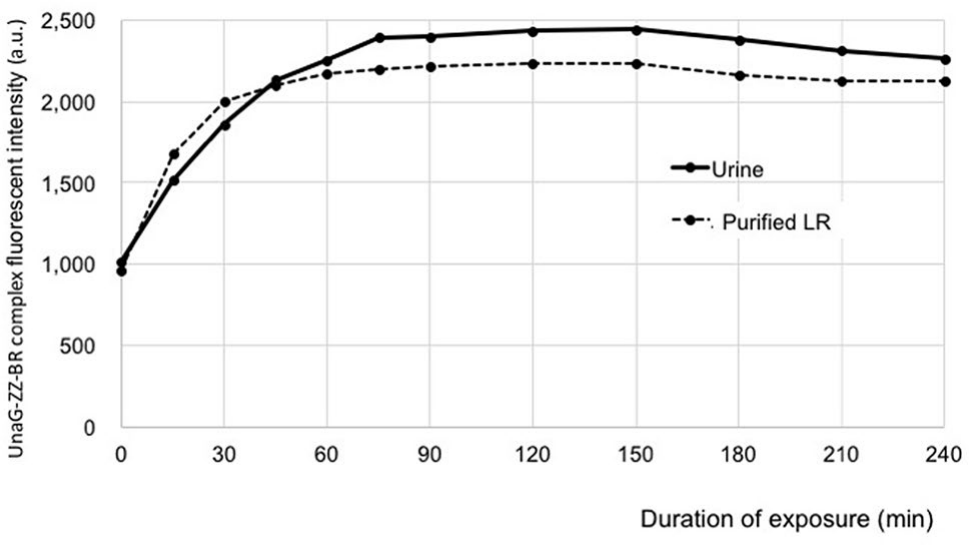
Fluorescence assay. Fluorescence intensity over time of 2 μmol/L purified LR and urine collected during phototherapy, each mixed with UnaG mixture (see Methods in the text) and then exposed to blue light. In both samples, the fluorescence intensity representing the UnaG–ZZ-BR complex increased with duration of exposure and plateaued at 60–75 min. LR, lumirubin; ZZ-BR, ZZ-bilirubin.

**Figure 2:**
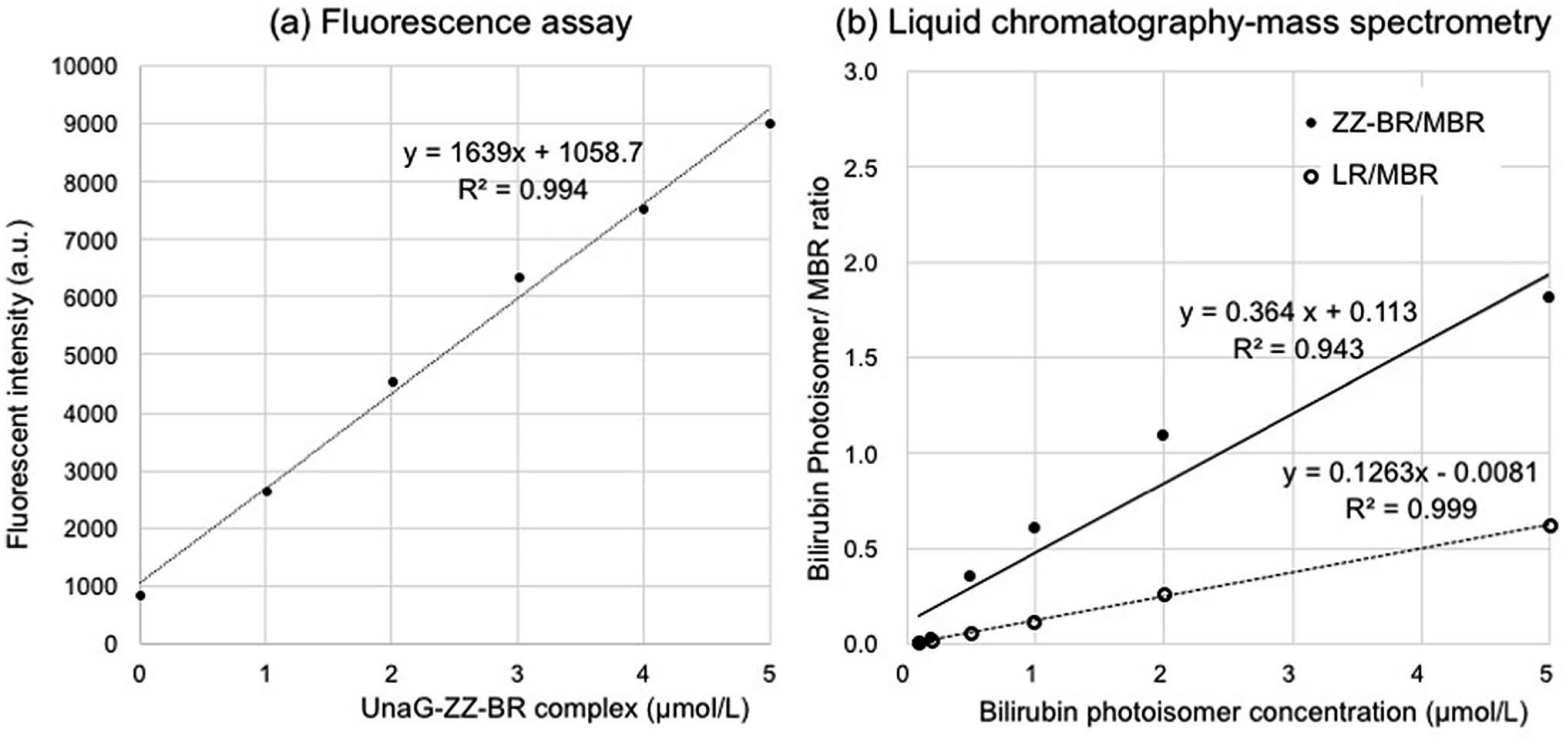
Bilirubin photoisomer calibration curves. (**a**) Fluorescence assay. Calibration curve was prepared from the fluorescence intensity of reference ZZ-BR adjusted to concentrations in the range of 0–5 μmol/L along with UnaG mixture (see Methods in the text). (**b**) Liquid chromatography–mass spectrometry. ZZ-BR and LR are expressed as the ratio of ion intensity to that of the internal standard, MBR. ZZ-BR, ZZ-bilirubin; LR, lumirubin; MBR, mesobilirubin.

The fluorescence intensity of both purified LR and neonatal urine increased over time, plateaued for a time, and then slowly decreased (Fig. 1). Using the calibration curve shown in Fig. 2a, the highest fluorescence intensity was converted to the UnaG-ZZ-BR complex concentration. A concentration of 2.0 µmol/L of purified LR was used for the FL, and the calculated concentration of UnaG-ZZ-BR complex was 0.72 µmol/L. As the urine samples were diluted fivefold prior to analysis, the final calculated concentration of UnaG-ZZ-BR complex in urine was 4.19 μmol/L. Unfortunately, it was not possible to determine the initial amount of LR in the urine samples using this method alone. Because this fluorescence assay is not a method to measure LR itself, but only to "estimate" the amount of LR that has been converted to UnaG-ZZ-BR complex.

### Liquid chromatography-mass spectrometry (LC–MS/MS)

#### Accuracy and stability of ZZ-BR and LR quantification

The HPLC retention times of LR, ZZ-BR, and mesobilirubin (MBR) were 2.00, 16.53, and 17.22 min, respectively. Employing MBR as an internal standard (ISTD), reliable quantification of LR (y = 0.1263x − 0.0081, *R*^2^ = 0.999) and ZZ-BR (y = 0.3639x + 0.1133, *R*^2^ = 0.943) was achieved over the concentration range 0.1–5.0 μmol/L (Fig. 2b). Details regarding validation of the LC–MS/MS method are as follows. For intra-day validation, 3 specimens representing 3 spiked concentration levels (ZZ-BR and LR were prepared at 0.1, 1, and 10 µmol/L) were analyzed 6 times in a single day. For inter-day validation, samples from 3 specimens were analyzed 6 times on each of 3 days over a 2-month period. The intra- and inter-day accuracy, precision, and recovery values were within the acceptable ranges for ZZ-BR and LR. For ZZ-BR, the intra- and inter-day accuracy values were within the range 0.10–1.21% and 0.59–0.98%, respectively, whereas the intra- and inter-day accuracy values for LR were within the range 0.12–7.78% and 0.63–5.66%, respectively. For ZZ-BR, the intra- and inter-day precision values were within the range 3.10–11.10% and 3.16–5.98%, respectively, whereas the intra- and inter-day precision values for LR were within the range 3.57–13.51% and 4.36–7.85%, respectively. Finally, the intra- and inter-day recovery values for ZZ-BR were within the range 98.79–99.90% and 99.02–99.41%, respectively, whereas the intra- and inter-day recovery values for LR were within the range 92.22–100.91% and 99.34–99.71%, respectively.

#### Reverse photoisomerization of purified LR and LR in urine

**Figure 3:**
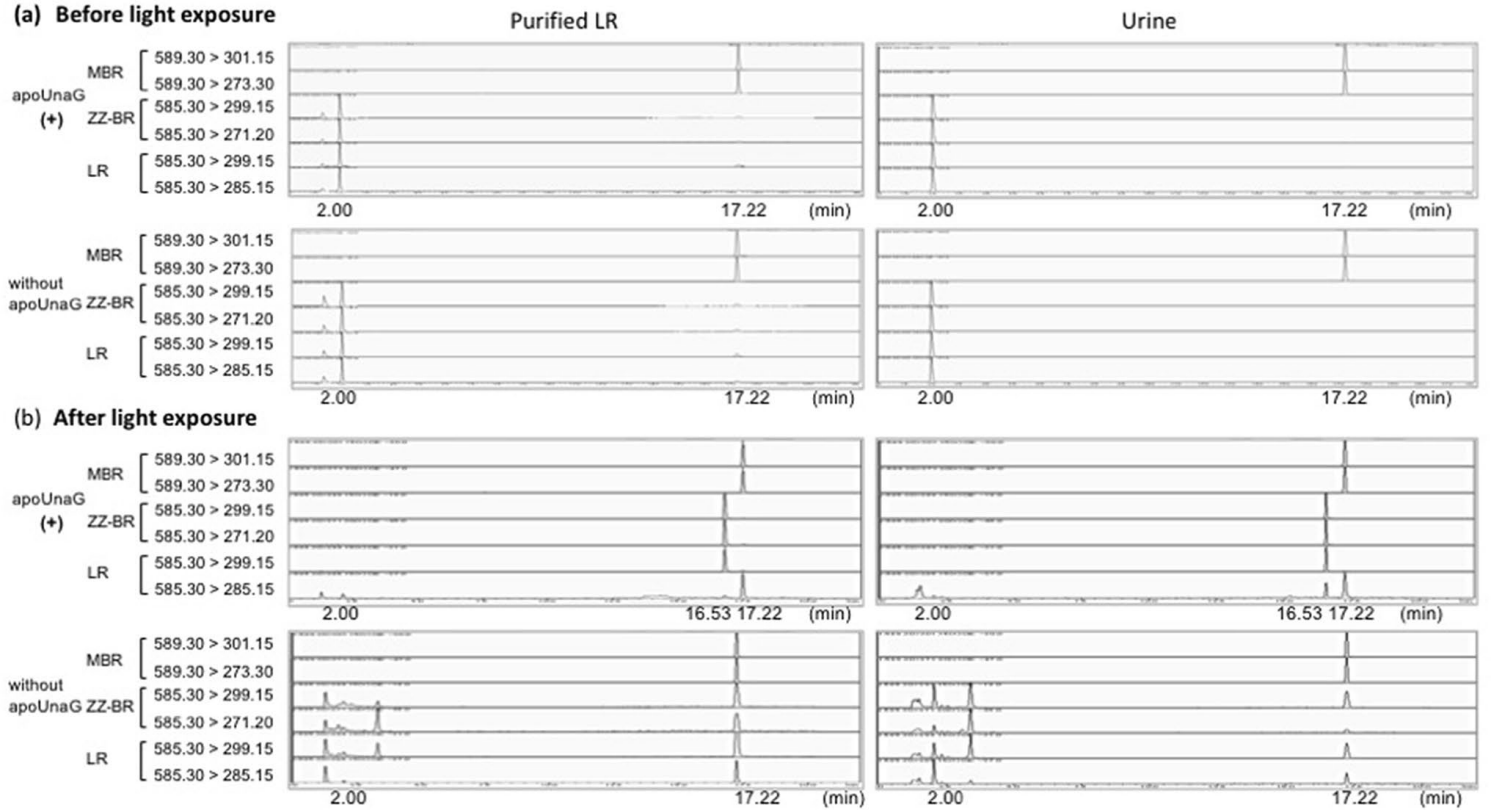
Reverse photoisomerization in LC–MS/MS. MRM spectra of purified LR and urine. The retention times of MBR, ZZ-BR, and LR were 17.22, 16.53, and 2.00 min, respectively. As MBR was used as an internal standard, a signal at 17.22 min was observed in all analyses. (**a**) Before blue light exposure. All MRM spectra showed the concentration of LR but not that of ZZ-BR. (**b**) After blue light exposure. ZZ-BR (16.53 min) appeared with apoUnaG in both purified LR and urine. Without apoUnaG, however, only LR decreased, and ZZ-BR was not detected, even after exposure to blue light. LC–MS/MS, liquid chromatography–mass spectrometry; MRM, multiple reaction monitoring; MBR, mesobilirubin; ZZ-BR, ZZ-bilirubin; LR, lumirubin.

The results of MRM (multiple reaction monitoring) LC–MS/MS analysis of ZZ-BR and LR are shown in Fig. 3. As MBR was used as an ISTD, a peak with a retention time of 17.22 min was observed in all MRM spectra. Before blue light exposure (Fig. 3a), all MRM spectra exhibited only an LR peak (2.00 min). After 90 min of blue light exposure (Fig. 3b) in the presence of apoUnaG, the LR peak in samples of both purified LR and urine almost disappeared, and a peak associated with ZZ-BR (16.53 min) appeared, indicating the photo-reconversion of LR to ZZ-BR. However, in the absence of apoUnaG, no indication of ZZ-BR formation was detected, even after light exposure.

The concentrations of LR and ZZ-BR were calculated using the calibration curve shown in Fig. 2b. In the presence of apoUnaG, the concentration of purified LR decreased markedly, from 0.46 to 0.06 μmol/L, and this was accompanied by an increase in the concentration of ZZ-BR to 0.12 μmol/L. By comparison, in the absence of apoUnaG, the concentration of LR decreased from 0.44 to 0.11 μmol/L, while the concentration of ZZ-BR was below the detection limit. Addition of apoUnaG to urine samples prior to blue light exposure resulted in a similar decrease in LR concentration from 4.50 to 0.47 μmol/L and concomitant increase in ZZ-BR concentration to 1.83 μmol/L after a 90-min light exposure. In the absence of apoUnaG, the concentration of LR in urine before light exposure was 4.68 μmol/L, and no ZZ-BR was detected (Fig. 3a); after light exposure, the LR concentration declined to 1.11 µmol/L, and the ZZ-BR concentration remained below the detection limit (Fig. 3b). These results indicate that the efficiency of reverse photoisomerization of LR to ZZ-BR in the presence of apoUnaG is 26–40%.

#### Reverse photoisomerization efficiency in urine

**Figure 4:**
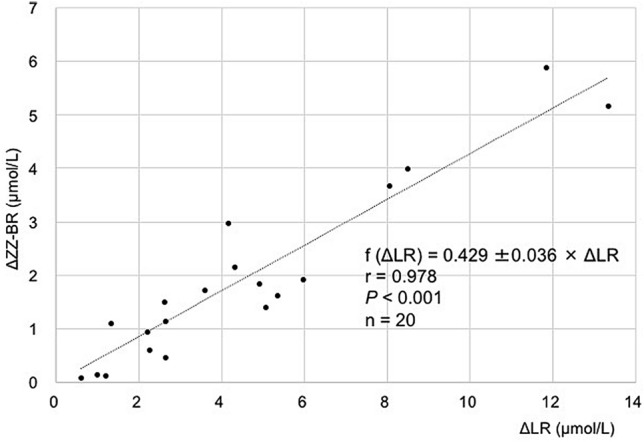
Correlation and efficiency of conversion of urinary LR to ZZ-B in the presence of apoUnaG following a 90-min blue light exposure. Single linear regression analysis of the increase in ZZ-BR concentration and decrease in LR concentration in urine samples during blue light exposure in the presence of apoUnaG. ΔZZ-BR is defined as the difference in ZZ-BR concentration before and after blue light exposure. ΔLR is defined as the absolute difference in LR concentration before and after blue light exposure. A strong positive correlation between ΔZZ-BR and ΔLR was observed. ZZ-BR, ZZ-bilirubin; LR, lumirubin.

A total of 20 urine samples obtained from 17 neonates were analyzed (Table 1). The gestational age ranged from 23.0 to 33.4 weeks (mean ± SD, standard deviation; 29.1 ± 3.8 weeks), and birth weight ranged from 500 to 1976 g (1190 ± 512 g). Serum total bilirubin concentration (TSB) were monitored pre- and during- PT. The mean ± SD of pre-PT TSB and during-PT TSB were 7.5 ± 3.1 and 6.8 ± 2.8 mg/dL, respectively. Urine samples were collected 51.0 ± 26.5 h after birth and 11 ± 4.4 h after initiation of PT. Each urine sample was subjected to LC–MS/MS analysis before and after reverse photoisomerization in the presence of apoUnaG. The urinary LR measured by LC–MS/MS before reconversion to the UnaG-ZZ-BR complex ranged from 1.46 to 14.91 (mean ± SD, 5.4 ± 3.9) μmol/L. Over a 90-min period of reverse photoisomerization, 82% (mean; SD, 16%) of the LR in urine was consumed. A plot of the increase in ZZ-BR (ΔZZ-BR) concentration versus the absolute decrease in LR (ΔLR) during reverse photoisomerization (Fig. 4) revealed a linear dependency (f [ΔLR] = 0.429 ± 0.036 × ΔLR; 95% CI 0.353–0.505; correlation coefficient, *r* = 0.946; 95% CI 0.867–0.979; *P* < .001, n = 20). These results are consistent with the determined efficiency of reverse photoisomerization of LR to ZZ-BR in a urine matrix of 43 ± 2% and concomitant LR degradation rate of 57 ± 2%.

**Table 1:** Subjects for Urinary bilirubin photoisomers measurement with apoUnaG.

No	Gestational age	Birth weight	Time after initiation of PT (after bitth)	Pre-PT TSB	During-PT TSB	Sampling time of blood & urine (after birth)	Urinary LR (LC–MS/MS)
	(wk)	(g)	(h)	(mg/dL)	(mg/dL)	(h)	(μmol/L)
1	23.0	586	14.5	4.5	4.4	34.3	14.91
2	23.2	626	12.5	6.2	5.4	33.8	1.88
3	23.4	501	19.8	6.4	5.5	52.3	2.64
4	25.1	792	7.0	6.2	5.0	35.3	3.54
5	25.2	724	9.3	4.5	3.0	31.5	5.35
6	27.1	964	16.7	4.8	3.3	72.3	5.60
7	28.3	974	5.3	4.3	3.3	31.2	3.13
8	29.5	500	11.3	5.4	5.2	21.3	7.71
9	29.5	1446	9.0	5.3	6.1	28.6	1.46
10	30.2	1494	9.0	4.7	5.1	32.0	2.05
11	32.0	1050	13.0	5.5	6.6	29.3	10.42
12	32.5	1940	11.5	14.2	10.0	108.4	14.36
13^a^	33.0	1904	5.8	12.0	12.5	84.9	2.45
14^a^			13.5	12.0	11.6	92.6	4.22
15^a^			21.5	12.0	10.5	100.6	1.64
16	33.2	1488	8.8	5.2	4.2	30.8	4.17
17	33.2	1976	7.5	7.8	8.2	37.0	4.91
18	33.3	1629	7.5	8.1	8.2	36.2	3.22
19^b^	33.4	1632	6.0	10.2	8.8	60.3	9.00
20 ^b^			10.7	10.2	8.5	67.0	4.93
Mean (SD)	29.1 (3.8)	1190 (512)	11.0 (4.4)	7.5 (3.1)	6.8 (2.8)	51.0 (26.5)	5.4 (3.9)

PT, phototherapy; TSB, total serum bilirubin; LR, lumirubin; LC–MS/MS, liquid chromatography-mass spectrometry.

^a^^,b^Multiple samples from the same patient.

## Discussion

The objective of PT for neonatal jaundice is to convert hydrophobic unconjugated bilirubin (ZZ-BR) to hydrophilic bilirubin photoisomers that are readily excreted via the bile and urine^10^. Several reports have described the excretion of hydrophilic unconjugated bilirubin, such as ZE-bilirubin and LR, via the urine^10,11^, and we also identified these bilirubin photoisomers by HPLC (Fig. S1 online). LR is reportedly the predominant bilirubin photoisomer excreted in humans^5^. Onishi and co-authors^10^ reported that following blue light exposure, the urine concentration of LR is equivalent to one-fifth of the concentration of LR in bile. However, samples in previous studies^5–7^ were assayed using HPLC, which is not available to ordinary clinicians for bedside testing. As such, the fate of LR during PT and its clinical significance have not been clarified.

In the present study, we developed a convenient FA for estimating the LR concentration. Using this method, we demonstrated the reverse photoisomerization of LR to ZZ-BR both in vitro and in patient-derived samples using LC–MS/MS, an established technique for the analysis of bilirubin photoisomers. We chose urine as the patient-derived sample because it can be collected non-invasively and because we speculated that monitoring urinary LR would aid in assessing the effectiveness of PT.

LR has been described as an irreversibly formed photoisomer of ZZ-BR in aqueous solution^11,12^. However, several studies have reported the reversion of a poorly defined bilirubin photoisomer—possibly LR—to ZZ-BR in oxygen-free chloroform upon light irradiation, albeit at low yield^7,13^. Consistent with these reports, blue light exposure of purified LR in aqueous solution did not yield detectable ZZ-BR in the present study. We hypothesized that the higher solvation energy of ZZ-BR compared with that of LR in aqueous solvent impedes the reverse photoisomerization of LR. In biological samples, the solubility of ZZ-BR is increased by binding to human serum albumin (HSA). Nevertheless, the presence of HSA (which has a moderate K_d_ of 87 nmol/L for ZZ-BR) in our artificial and patient-derived urine samples did not appear to affect the LR/ZZ-BR equilibrium. We speculated that the presence of the ZZ-BR–specific protein apoUnaG^1^, which has a reported K_d_ of 98 pmol/L (three orders of magnitude greater than that of HSA), reduces the energy penalty associated with the reverse photoisomerization of LR by lowering the concentration of fully solvated ZZ-BR in solution. Indeed, in the presence of apoUnaG, reverse photoisomerization of LR to ZZ-BR was observed in both the artificial and patient-derived samples, despite the presence of oxygen in each solution.

In addition to the high specificity and affinity of UnaG with respect to ZZ-BR, the ZZ-BR–UnaG complex reportedly exhibits concentration-dependent fluorescence^1^.

ZZ-BR is composed of four pyrrole rings (rings A/B/C/D), and is also represented as a molecule bound by the endo-vinyl dipyrrinone (rings A/B) and the exo-vinyl dipyrrinone (rings C/D) (see Fig. S2 online). The atomic structure of UnaG bound to ZZ-BR has been elucidated^1^. Bound ZZ-BR is inserted within the barrel of UnaG in a central internal cavity. The exo-vinyl dipyrrinone moiety (rings C/D) and the B-ring propionate are accommodated deep in the cavity, whereas the endo-vinyl dipyrrinone moiety (rings A/B) and the C-ring propionate are positioned near the entrance. Our co-authors reported that even bilverdin, which only lost one H^+^ of the endo-vinyl dipyrrinone that composes ZZ-BR, does not bind to UnaG^1^. Thus, considering the crystal structure of UnaG in complex with ZZ-BR, lumirubin, partially cyclized of the endovinyl dipyrrinone, would no longer be allowed to bind to UnaG.

It should be noted that the reconversion of lumirubin to ZZ-BR is exclusively due to the action of UnaG along with blue light, and this phenomenon cannot be captured in the absence of UnaG. Thus, since humans do not have the fluorescent protein apoUnaG, there would be no need to consider the influence of reconversion, even during PT.

While the mortality rate of preterm infants is improving, low-bilirubin kernicterus is rare, but refractory cause of bilirubin neurotoxicity in them^14^. In a given infant with low-bilirubin kernicterus, both an albumin problem and a vulnerable neuronal pool are evident^15^. In addition to those causes, we hypothesized that the earlier in life and the more immature the infant, the more bilirubin accumulates in the tissues, which may also be involved in the low-bilirubin kernicterus. Therefore, considering the need to evaluate bilirubin excretion, especially in preterm infants, we developed a simple detection method for bilirubin photoisomers excreted in the urine during PT.

Considering further optimization of the reverse photoisomerization assay as well as of the fluorescence-based assay for quantifying bilirubin photoisomers, the method described herein could provide rapid, non-invasive feedback to assess the efficacy of PT in the future.

The general conclusions of this study are limited by the small number of infants enrolled. For example, the potential negative effect of the excretion of thiol-containing medications such as L-cysteine on the stability of bilirubin and LR in urine has not been assessed. Moreover, we speculate that the < 50% efficiency of LR reconversion to ZZ-BR using this method was due to the effects of light. Photo-degradation^16^, photo-oxidation^17^, or photo-bleaching of these analytes or their complexes during reverse photoisomerization due to light and oxygen exposure have not been adequately assessed.

## Methods

### Reagents and preparation

#### Bilirubin (ZZ-BR) solution

Bilirubin reagent was used without further purification because the present examination was performed by LC–MS/MS (see Fig. S3 online). A total of 2 mg of bilirubin (98%) as ZZ-BR was dissolved in 2 mL of 0.1 mol/L NaOH, immediately neutralized with 1 mL of 0.1 mol/L phosphoric acid, and mixed with 7 mL of rabbit serum albumin (RSA; Sigma-Aldrich, St. Louis, MO, USA). The ZZ-B concentration was measured using a BL-300 2-wavelength spectrophotometer (TOITU Co., LTD, Tokyo, Japan) (455 nm, 575 nm), and the bilirubin solution was then diluted with PBS (0.1 mol/L, pH 7.2) to the required concentration. The above reagents except RSA were purchased from FUJIFILM Wako Pure Chemical Co. (Osaka, Japan).

#### UnaG mixture preparation

The apoUnaG used in this study is a fluorescent protein isolated from Japanese eel muscle as previously reported^1^. The “UnaG mixture” for the FA was prepared using apoUnaG, HSA, and ascorbic acid at a 1:1:1 (v/v/v) ratio, so that the final concentrations were 5 µmol/L, 0.01%, and 0.1%, respectively (see Supplement Methods and Figs. S4-S6 for the reasons for the addition of each substance). HSA (5% I.V.) and L ( +)-ascorbic acid were purchased from Japan Blood Products Organization (Tokyo, Japan) and FUJIFILM Wako Pure Chemical Co., respectively. Ascorbic acid was added to reduce the effects of photo-oxidation.

#### Purified LR preparation

LR was prepared for LC–MS/MS using preparative thin-layer chromatography as described previously^8,9^, with some modifications. Our method and those reported in the literature differed with regard to the light illuminator used for converting ZZ-BR to LR and the procedure after scraping the yellow band corresponding to LR from the silica gel plate. The light illuminator was a custom-built P4630 bi-color LED unit (Ushio Inc., Tokyo, Japan). The wavelength range (with peak emissions; color) was 440–590 nm (bi-modal peak, 455, 515; blue, green). The energy of light intensity was 9.0 mW/cm^2^ at a distance of 4 cm above the petri dish (4 cm diameter) containing the ZZ-BR solution, and the ZZ-BR solution was exposed for 90 min. Spectral intensity was determined by integrating each spectrum as a function of wavelength measured using a calibrated HR4000 spectrometer (Ocean Optics, Dunedin, FL). The yellow band corresponding to LR scraped from the silica gel plate was extracted with methanol (LC/MS-grade, FUJIFILM Wako Pure Chemical Co.). The extracted LR solution was centrifuged for 10 min at 2600×*g* (KUBOTA 2800, Kubota Co., Tokyo, Japan), and the supernatant was purified by solid-phase extraction using a Sep-Pak C18 Plus Short Cartridge (Waters, Milford, MA). The concentration of purified LR was determined spectrophotometrically at 453 nm (SpectraMax L&M2, Molecular Devices, LLC, San Jose, CA). The previously reported LR molar absorption coefficient was 33,000 mol^−1^ dm^3^ cm^−1^^12^.

#### ISTD preparation

The ISTD for LC–MS/MS was prepared by dissolving MBR (Frontier Scientific, Logan, UT) in dimethylsulfoxide (Thermo Fisher Scientific, Waltham, MA) at a concentration of 50 mmol/L. The ISTD was then stored at − 80 °C in 10-μL aliquots. Immediately before use, the ISTD was diluted with dimethylsulfoxide to a final concentration of 1 μmol/L.

### Subjects

The subjects enrolled in this study included 8 female and 9 male premature infants born before 34 weeks of gestation who were admitted to the neonatal intensive care unit of Nara Medical University Hospital, Nara, Japan, between December 2019 and March 2021. Except during PT, each neonate was housed in an incubator shielded from light by a shade cover. Infants with hemolytic diseases, infections, elevated conjugated bilirubin levels, gastrointestinal diseases, intraventricular hemorrhage, or congenital anomalies were excluded.

This study was approved by the Nara Medical University ethics committee (approval no.1033) and performed in accordance with relevant guideline. And informed consent was obtained from the parents of each infant. Decisions regarding the initiation and termination of PT were based on standard clinical criteria^18^. PT was administered continuously using a blue light–emitting diode PT system (neoBlue®, Natas Medical, San Carlos, CA) operated in “high mode” (length 450–475 nm/ intensity 35 µW/ cm^2^/ nm).

### Urine samples

To collect urine, several small cotton balls were placed near the external genitalia in the infant’s diaper. Immediately after collection, cotton balls were then squeezed to transfer the urine to a micro-tube (ST-0150R, INA, OPTICA Co., Ltd., Osaka, Japan) and frozen at − 80 °C until use. Urine samples were centrifuged for 10 min at 2600×*g* at 4 °C. The supernatants were diluted 3- or 5-fold with PBS, and samples analyzed by LC–MS/MS were filtered through a syringe filter (Minisart RC4, 0.45 µm RC-membrane, Sartorius Stedim Lab Ltd., Stonehouse, UK).

### Blood samples

Concentrations of TSB were determined from capillary blood (50 µL) collected from pricking the heel into sodium heparinized micro-hematocrit capillary tubes (Becton, Dickinson and Company, Franklin Lakes, NJ, USA) at pre- and during- PT. Serum was immediately separated by centrifugation for 5 min at 11,800×g (Kubota Corp., Tokyo, Japan) and then TSB was measured using 2-wavelength (455 nm, 575 nm) spectrophotometry (BL-300, TOITU Co., Tokyo, Japan). Blood samples were collected within 2 h of the initiation of PT for pre-PT TSB and within 1 h after urine collection for during-PT TSB.

### FA

A black microplate (Microtest™ 96-well assay plate, black, flat bottom, BD Biosciences, Franklin Lakes, NJ) was prepared, and 50 µL of sample (reference ZZ-BR, urine, or purified LR) was added to 150 µL of the UnaG mixture described above. The resulting 200-µL reaction mixture was pipetted into each well of the microplate. The fluorescence intensity of each well was assayed spectrophotometrically at 37 °C with fluorescence filters for excitation and emission wavelengths of 498 and 527 nm, respectively. For calibration curve generation, the ZZ-BR solution was diluted with PBS to concentrations of 0, 1, 2, 3, 4, and 5 µmol/L and analyzed as described above. Before light exposure, the calibration curve was drawn as UnaG-bound ZZ-BR (UnaG-ZZ-BR complex).

For the FA, a thawed urine sample (from a male patient born at 30 weeks of gestation and sampled at 23 h after initiation of PT) and 2 µmol/L purified LR were exposed to blue-light LED irradiation (P4630-blue LED unit, Ushio Inc., wavelength range 420–520 nm, with peak emission at 450 nm, 10.5 mW/cm^2^ at a distance of 4 cm above the microplate). Samples were assayed in duplicate every 15 min (until 90 min) or 30 min (over 90 min). The ZZ-B–UnaG complex concentration was defined as the highest fluorescence intensity during the exposure time as determined from the calibration curve (Fig. 2a).

### LC–MS/MS

#### Apparatus and chromatographic conditions

Chromatography was performed based on the method described by Jasprova and co-authors^9^, with some modifications. Analyses were performed using a gradient elution reversed-phase HPLC system (LC-20AD, Shimadzu Co., Kyoto, Japan) equipped with a Poroshell 120 EC-C18 column (3.0 × 100 mm; 2.7 μm, Agilent, CA, USA) and operated at a flow rate of 0.4 mL/min. LC–MS/MS was carried out using a Shimadzu LCMS-8045 triple quadrupole mass spectrometer equipped with a heated electrospray ionization probe. MBR, ZZ-BR, and LR were analyzed using collision-induced dissociation with selected reaction monitoring. Argon was used as the collision gas at a pressure of 230 kPa. For gradient elution, mobile phase A was 0.1% formic acid in ultrapure water, and mobile phase B was 0.1% formic acid in acetonitrile. The linear and stepwise gradient was programmed as follows: 0–3 min, 50% solvent B; 3–13 min, 50% to 100% solvent B; 13–18 min, 100% solvent B; 18–18.1 min: 100% to 50% solvent B; 18.1–22 min: 50% solvent B. The injection volume was 10 μL, and each sample was analyzed twice. The column temperature was 30 °C. The selected reaction monitoring transitions (corresponding collision energies) were as follows: for ZZ-B *m/z* 585.3–299.15 (18 V; quantifier) and *m/z* 585.3–271.20 (45 V; qualifier); for LR *m/z* 585.3–299.15 (31 V; quantifier) and *m/z* 585.3–285.15 (27 V; qualifier); and for MBR *m/z* 589.3–301.1(20 V; quantifier) and *m/z* 589.3–273.2 (47 V; qualifier). Data acquisition and integration were performed using Shimadzu Lab Solutions software (Shimadzu Co., Kyoto, Japan).

#### Validation

The LC–MS/MS method was validated in accordance with the Guidance for Industry, Bioanalytical Method Validation protocol^19^. Three different doses (0.1, 1, and 10 μmol/L) of ZZ-BR and purified LR were each assayed six times a day for 3 days to validate the method. Working solutions ranging in concentration from 0.1 to 10 μmol/L in 0.4% ascorbic acid as an antioxidant were prepared. The accuracy of the analytical method refers to the closeness of the mean test result obtained using the method to the actual value of the analyte: accuracy (%) = 100 × (mean of six determinations − actual value)/actual value. The precision of the analytical method refers to the closeness of individual measures of an analyte when the procedure is applied repeatedly to multiple aliquots of a single homogenous sample of biological matrix: precision (%) = 100 × standard deviation of six determinations/mean of six determinations. The recovery of an analyte in an assay refers to the detector response obtained from the amount of analyte added to and extracted from the biological matrix. This was compared with the detector response obtained for true concentrations of the analyte in the solvent: recovery (%) = 100 − accuracy.

#### Calibration curve preparation

ZZ-BR solution and purified LR diluted in 0.4% ascorbic acid were mixed in equal amounts and further diluted in PBS to achieve final concentrations of 0.1, 0.2, 0.5, 1, 2, and 5 μmol/L. Immediately prior to LC–MS/MS assay, MBR was thawed and diluted in 0.04% HSA to 5 μmol/L, added to each mixture to a final concentration of 1 μmol/L, and vortexed thoroughly. All samples for preparation of the calibration curve were assayed by LC–MS/MS in 10-μL volumes in duplicate, and the calibration curves were prepared for ZZ-BR and LR, expressed as MBR (ISTD) ratios.

#### Photoconversion by artificial blue light exposure

To verify the reconversion of LR to ZZ-BR in the presence of apoUnaG after artificial blue light exposure, four aliquots were prepared for purified LR and a urine sample obtained from a male infant born at 33 weeks of gestation and sampled 7.5 h after the initiation of PT. For the first two aliquots containing 50 μL each of sample (purified LR or urine), 0.05% HSA, 0.5% ascorbic acid, and 5 μmol/L MBR were prepared. Next, 50 μL of 25 μmol/L apoUnaG was added to one aliquot and 50 μL of PBS to the other as a control. These samples were directly analyzed by LC–MS/MS for LR before artificial light exposure. The remaining two aliquots were prepared for blue light exposure and consisted of 50 μL of 25 μmol/L apoUnaG or PBS, 50 μL of 0.05% HSA, 50 μL of 0.5% ascorbic acid, and 50 μL of sample (purified LR or urine). Each sample was poured separately into wells of a black microplate of the same type used for FAs. The microplate was then exposed to the same blue LED light (P4630-blue LED unit) and at the same emission as used for FAs. After 90 min of exposure, 160 μL of the mixture was withdrawn from each well and mixed with 40 μL of 5 μmol/L MBR for subsequent LC–MS/MS analysis of LR and ZZ-BR.

#### Correlation between urinary ZZ-BR after blue light exposure in the presence of apoUnaG and urine LR

Urine samples were collected from neonates during the PT and examined. The purpose of this study was to determine the correlation between the concentration of LR excreted in the urine and the concentration of ZZ-BR artificially reconverted after blue light exposure. LR and ZZ-BR concentrations were measured as described in the previous section. Urine LR (pre-LR) and ZZ-BR (pre-ZZ-BR) concentrations were determined before blue light exposure, and LR (post-LR) and ZZ-BR (post-ZZ-BR) concentrations were determined after blue light exposure. ΔLR was defined as the molar concentration of pre-LR minus post-LR (μmol/L), whereas ΔZZ-BR was defined as the molar concentration of post–ZZ-BR minus pre–ZZ-BR (μmol/L). The correlations between concentrations were then determined.

### Statistical analysis

Linear regression analyses were performed using StatFlex software, ver. 6 (Artech Co., Osaka, Japan), and Excel Statistics (Excel® 2013, Microsoft Japan Co., Ltd., Tokyo, Japan).

## Supplementary Information

Supplementary Information.

## Data Availability

All data generated or analyzed during this study are included in this published article.
