# Supplementary material for: Noninvasive monitoring of bilirubin photoisomer excretion during phototherapy
Source: Sci Rep. 2022 Jul 12;12:11798. doi: 10.1038/s41598-022-16180-9 (PMC9276810; doi:10.1038/s41598-022-16180-9)
Supplement: Supplementary file 1 — Supplementary Information. [file 41598_2022_16180_MOESM1_ESM.pdf]

Supplementary Information

TITLE

Noninvasive Monitoring of Bilirubin Photoisomer Excretion during Phototherapy

AUTHORS

Yumiko Uchida, M.D. Ph.D.; Yukihiro Takahashi, M.D. Ph.D.; Yukihiro Morimoto, Ph.D.; Peter Greimel, Ph.D.; Asako Tosaki, Ph.D. [4]; Akiko Kumagai, Ph.D.; Toshiya Nishikubo, M.D. Ph.D.; Atsushi Miyawaki, M.D. Ph.D.

SUPPLEMENTALY METHODS

Preliminary experiments on measurements

ApoUnaG concentration on the assay

The concentration of apoUnaG sufficient for the assay was preliminarily examined. Urine samples were collected three times a day from day 0 to 7 after birth from preterm infants born in-hospital between 22 and 33 weeks gestation, after consent was obtained from the parents. Of these, 66 preterm infants treated with phototherapy were included. The median of gestational age was 30.2 (standard error; SE 2.6) weeks and of birth weight was 1,275 (SE 468) g. Urine samples diluted 3-fold in PBS in the presence of a final concentration of apoUnaG 10 μmol/L were blue light exposed and fluorescence assayed. The UnaG-ZZ-BR complex was maximal at 14.5 ± 1.6 hours after phototherapy (median

± SE). The distribution of maximum UnaG-ZZ-BR complex values in each case is shown in Fig. S3 online. And The median of the maximum UnaG-ZZ-BR complex was 4.48 (SE 0.60) μmol/L. And the highest value is 20.96 μmol/L. ApoUnaG and ZZ-BR bind at a 1:1 (molar ratio) [1 in the text]. Under these conditions, theoretically up to 30 μmol/L of ZZ-BR should be able to bind apoUnaG. On the other hand, since most of them are distributed between 3 and 7 μmol/L. Therefore, for the examinations presented in the text, the final apoUnaG concentration was prepared to 5 μmol/L and the urine samples were diluted 3 to 5-fold in PBS.

The related contents of parameters optimization for the assay

For optimization of the parameters required for the assay, we added HSA and ascorbic acid in the reaction solution.

HSA

As the HSA concentration increases, more photoproduct is produced until a [HSA]/[BR] of 1.5 is reached. At higher concentrations of HSA, the amount of photoproduct decreases, perhaps because the binding of bilirubin decreases due to protein aggregation [Sailofsky & Brown]. So, we examined whether final concentration of 1.5 μmol/L (0.01%) or 15 μmol/L (0.1%) HSA concentrations would better detect urinary UnaG-Bil complex by fluorescence assay with blue light exposure in the

presence of apoUnaG. Urine samples from a girl born at 25 weeks of gestation were examined. As shown in Fig. S4 online, when 1.5 μmol/L HSA was added, urine samples collected during phototherapy exhibited higher urinary UnaG-Bil complex contents. For this reason, we decided to use 1.5 μmol/L HSA (0.01%) as one component of the "UnaG mixure".

Ascorbic acid

Another component, ascorbic acid, was added to reduce photo-oxidation caused by blue light exposure. Pooled sera (without phototherapy) collected from preterm infants and bilirubin reagent were prepared at 0, 12.5, 25, 37.5, 50, and 62.5 μg/dL, respectively. Various concentrations of ascorbic acid (final concentrations 0, 0.02, 0.05, 0.1%; w/v) were added to these samples and then exposed to blue light with the final concentration of 2 μmol/L apoUnaG and 0.1% HSA. The antioxidant effect of ascorbic acid was expressed as the ratio of fluorescence intensity at 0 min to that at 240 min of exposure. The fluorescence intensity ratio that showed significant differences in both sera and bilirubin reagent was "0% vs. 0.1%" final concentration of ascorbic acid (Fig. S5 online). Statistical analysis was by t-test using Excel Statistics (Excel® 2013, Microsoft Japan Co., Ltd., Tokyo, Japan). As a result, we determined to add ascorbic acid to the "UnaG mixure" at a final concentration of 0.1%.

SUPPLEMENTALY REFERENCE

Sailofsky, B. M. & Brown, G.R. Solvent effects on the photoisomerization of bilirubin. *Can. J. Chem.* **65**, 1908-1916 (1987)

SUPPLEMENTARY FIGURE

Fig. S1 HPLC chromatogram of bilirubin photoisomers in urine

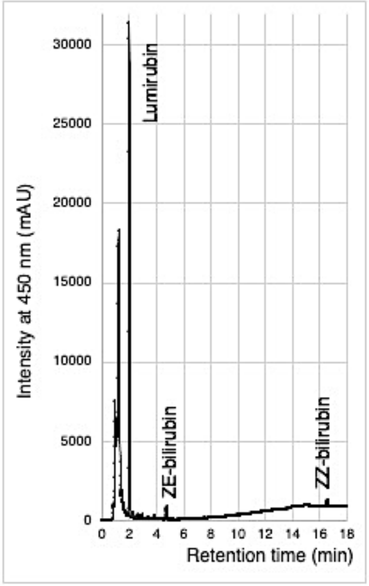

HPLC chromatogram of urine from a neonate born at 28 weeks of gestation, collected 10 hours after the initiation of phototherapy. The chromatogram with a photo diode array detector (SPD-M20A, Shimadzu Co., Kyoto, Japan) at 450 nm. Peaks of lumirubin, ZE-bilirubin, and ZZ-bilirubin were observed at retention times of 2.00, 4.75, and 16.85 min, respectively.

Fig. S2 Structures of bilirubin and related compounds

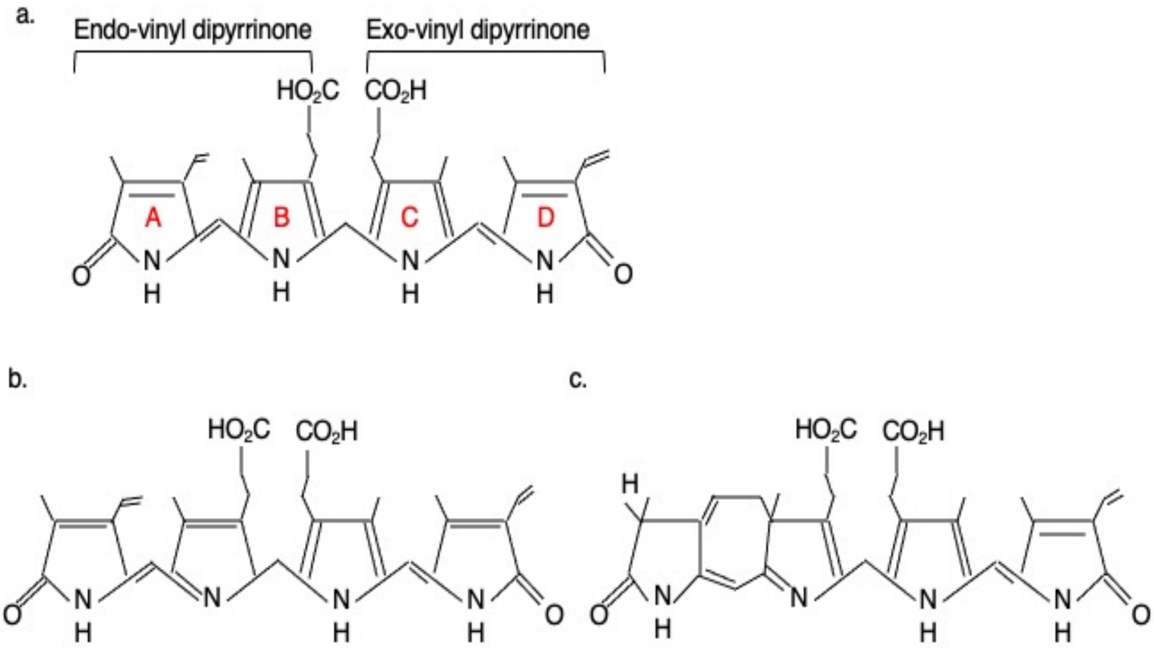

(a) ZZ-bilirubin. (b) Biliverdin. It is formed by oxidation of bilirubin. (c) Lumirubin.

It is one of photoisomers, a partial cyclization of ZZ-bilirubin.

Fig. S3 ZZ-bilirubin of detection by LC-MS/MS (bilirubin reagent)

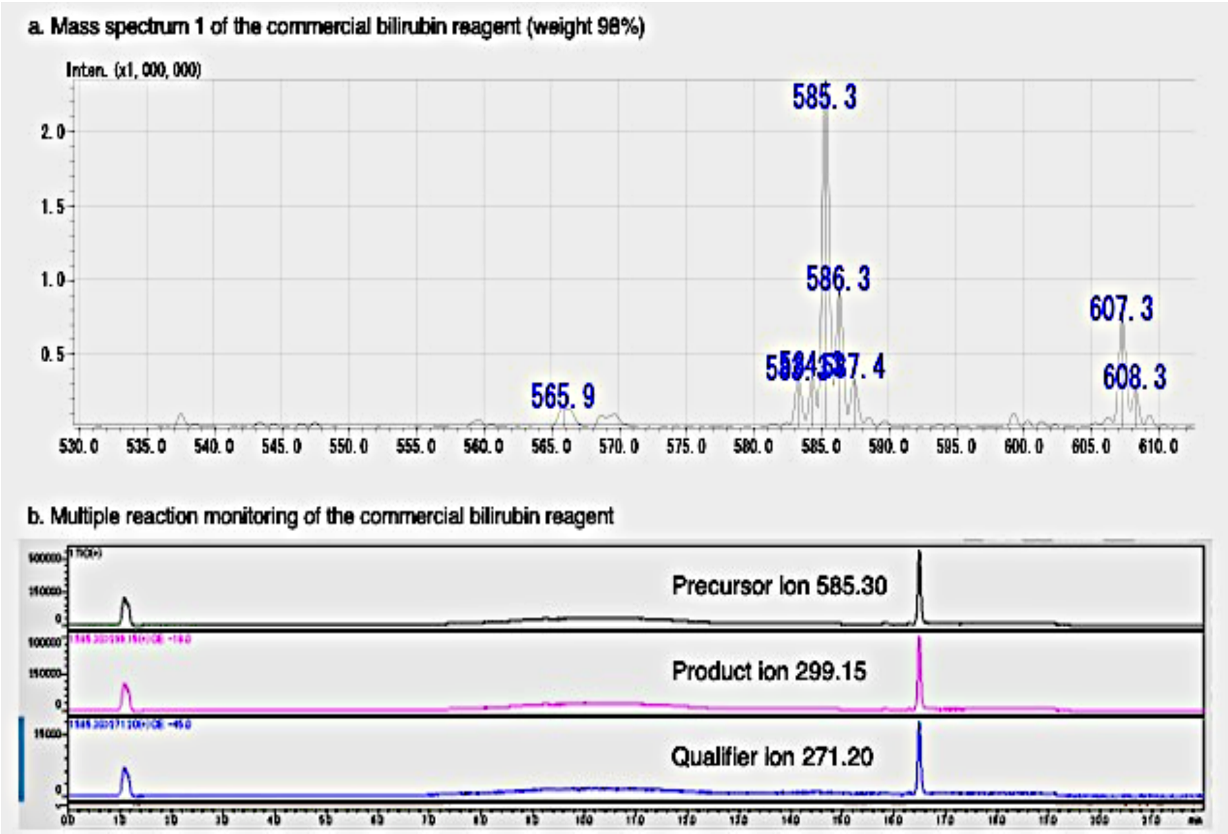

(a) Mass spectrum of the bilirubin reagent. Various ion peaks other than 585.3, the precursor ion of the target ZZ-bilirubin were observed. (b) Multiple reaction monitoring. The precursor, product, and qualifier ion peaks specific to ZZ-bilirubin were observed at at retention times of 16.58 min.

Fig. S4 Range of the maximum urinary UnaG-ZZ-BR complex by fluorescence assay

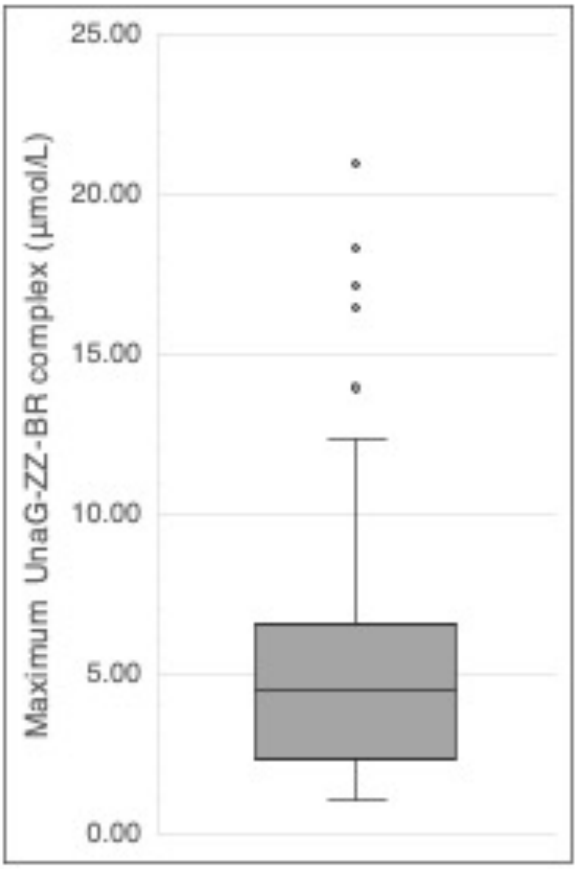

median (SE) = 4.48 (0.60) μmol/L
n= 66

Distribution of the maximum urinary UnaG-ZZ-BR complex contents presented during the first 7 days of life in 66 preterm infants. Fluorescence assay with blue light exposure on the reaction solution of 3-fold diluted urine with the final concentrations of 10 μmol/L apoUnaG, 0.1% ascorbic acid, and 0.01% HSA. The highest maximum urinary UnaG-ZZ-BR complex content was 20.96 μmol/L.

Abbreviations: SE, standard error; ZZ-BR, ZZ-bilirubin; HSA, human serum albumin

Fig. S5 Comparison of urinary UnaG-ZZ-BR complex contents at different HSA concentrations

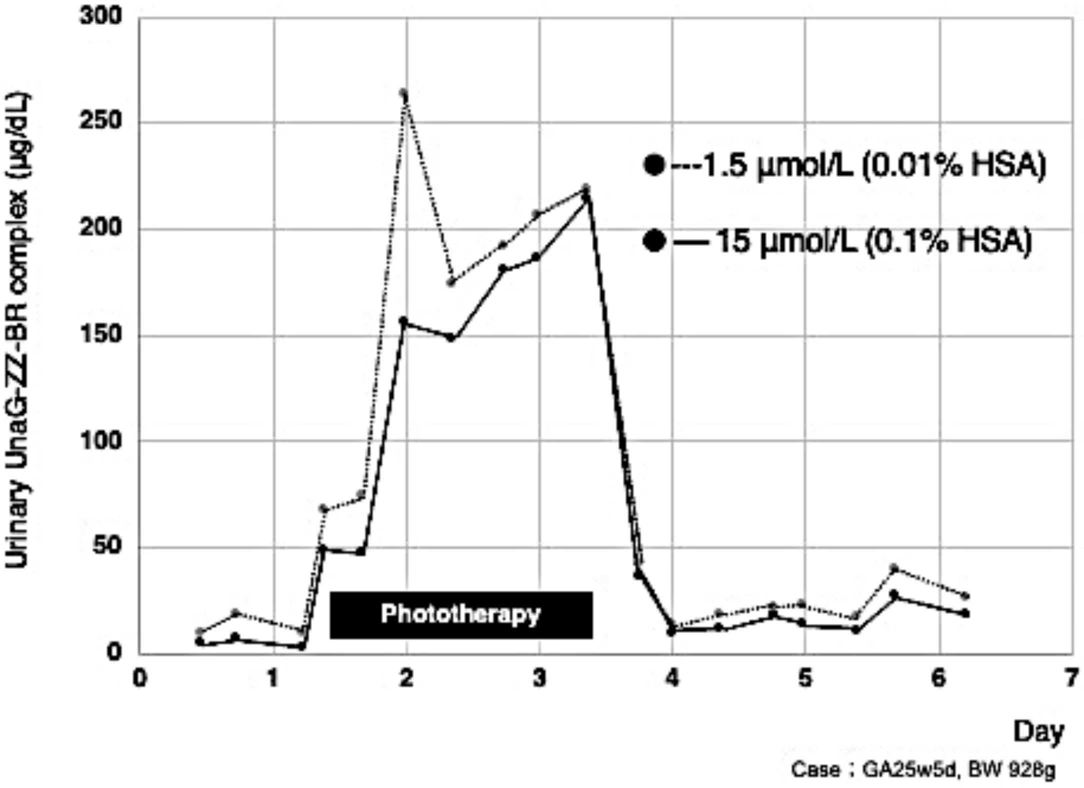

Urine samples collected from one preterm infant were analyzed for fluorescence at two different HSA concentrations (the other conditions are the same as in the text). The UnaG-ZZ-BR complex contents were higher in 0.01% HSA than in 0.1% HSA, especially in samples during phototherapy. (UnaG-ZZ-BR complex; 100 µg/dL = 1.71 µmol/L)

Abbreviations: HSA, human serum albumin; ZZ-BR, ZZ-bilirubin

Fig. S6 The antioxidant effect of ascorbic acid

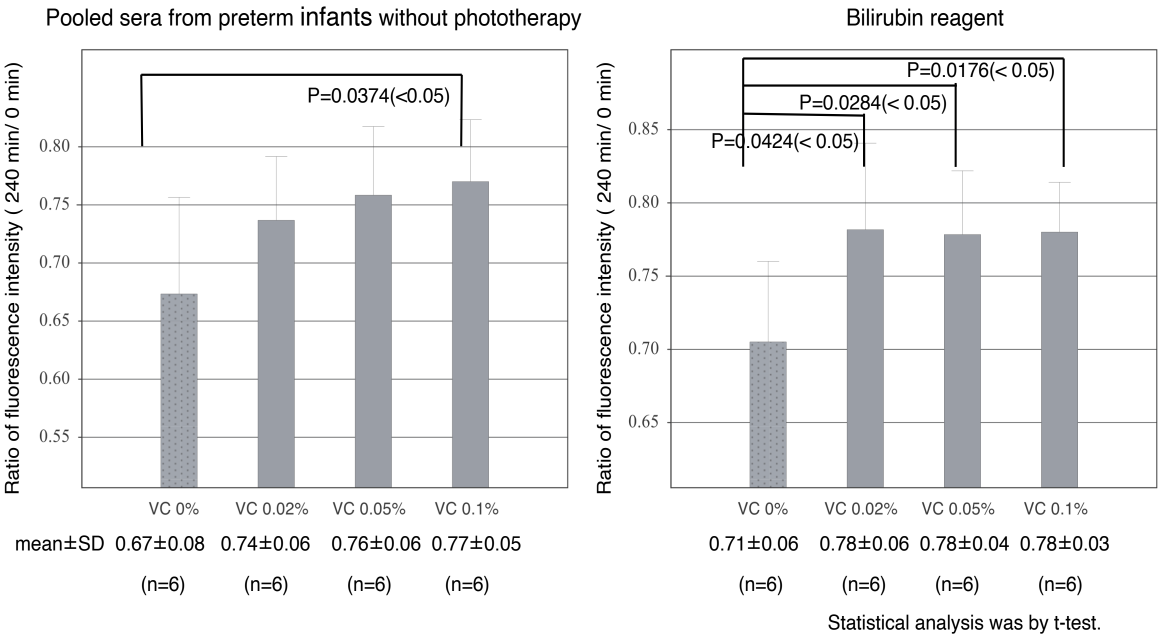

Ratio of fluorescence intensity of the sample before blue light exposure (0 min) to that after 240 min of exposure. In both sera and bilirubin reagents, the absence of ascorbic acid (0% VC) resulted in a significant loss of fluorescence intensity compared to those with 0.1% added.

Abbreviations: VC, vitamin C (= ascorbic acid)
